# Impaired placental mitophagy and oxidative stress are associated with dysregulated BNIP3 in preeclampsia

**DOI:** 10.1038/s41598-021-99837-1

**Published:** 2021-10-14

**Authors:** Xiaobo Zhou, Xue Zhao, Wei Zhou, Hongbo Qi, Hua Zhang, Ting-li Han, Philip Baker

**Affiliations:** 1 Department of Obstetrics and Gynecology, The First Affiliated Hospital of Chongqing Medical University, 1 Youyi Road, Yuzhong, Chongqing, 400016 China; 2 Chongqing Key Laboratory of Maternal and Fetal Medicine, Chongqing, 400016 China; 3 Joint International Research Laboratory of Reproduction and Development of Chinese Ministry of Education, Chongqing Medical University, Chongqing, 400016 China; 4 Liggins Institute, University of Auckland, Auckland, 1023 New Zealand; 5 The Center for Reproductive Medicine, Obstetrics and Gynecology Department, The Second Affiliated Hospital of Chongqing Medical University, Chongqing, 400010 China; 6 College of Medicine, Biological Sciences and Psychology, University of Leicester, Leicester, LE1 9HN UK; 7 Department of Obstetrics, Chongqing Health Center for Women and Children, No. 120 Longshan Road, Yubei District, Chongqing, 400021 China

**Keywords:** Pathogenesis, Reproductive disorders

## Abstract

Preeclampsia (PE) is a severe multisystem pregnancy complication characterized by gestational hypertension and proteinuria. Bcl-2/adenovirus E1B 19-kDa interacting protein 3 (BNIP3) is a mediator of mitophagy and has been proven to be associated with PE, but the mechanism is not well understood. This study aimed to investigate the role of BNIP3 in PE. Placentae from preeclamptic and normal pregnancies were analyzed by western-blot and transmission electron microscopy to quantify the level of BNIP3 expression and observe the organelle morphologies. Trophoblast cells with knockdown BNIP3 were analyzed by western-blot, immunofluorescence, flow cytometry, migration and invasion assays. BNIP3 expression was suppressed in PE patients. Impaired autophagy and increased mitochondrial damage were observed in PE placentae when compared with normal placentae. Suppression of BNIP3 inhibited Beclin-1 expression and reduced the transformation of LC3-I to LC3-II. In the knockdown BNIP3 group, p62 was overexpressed, ROS accumulated and the apoptotic process was elevated under oxidative stress condition. The knockdown of BNIP3 reduced the colocalization of GFP-LC3 and mitochondria. The findings of this study suggest that dysregulated BNIP3 is associated with impaired mitophagy, oxidative stress, and apoptosis in PE. The study provides new insights into the role of BNIP3 in the pathophysiology of PE.

## Introduction

Preeclampsia (PE) is a life-threatening pregnancy-specific syndrome characterized by gestational hypertension and proteinuria. PE occurs after 20 weeks of gestation and affects about 2–8% of pregnancies all over the world^1–3^. PE remains a high cause of maternal and fetal morbidity and mortality worldwide^1^. PE can also increase the susceptibility to cardiovascular and cerebrovascular diseases, such as hypertension, kidney diseases, coronary heart diseases, stroke, and metabolic syndrome in later life of both mothers and their offspring^4^. There is no single test that can predict who will develop PE, therefore there is no way to completely prevent it. Early-onset PE is thought to be mediated by the placenta with shallow extravillous trophoblasts invasion. The main pathophysiological feature is incomplete transformation of the uterine spiral arteries, resulting in hypoperfusion of the placenta and reduced nutrient supply to the fetus^5–7^. It is characterized by excessive cell death, including apoptosis and autophagy in the placenta^8–10^. In contrast, late-onset PE is thought to be the manifestation of a mismatch between the normal maternal supply and the metabolic demands of the growing placenta and fetus. There are well-known associations between late-onset PE and maternal genetic predisposition to cardiovascular and metabolic disease, and a high body mass index (BMI). The mechanisms and pathways of autophagy in placental cells, however, remain unknown to date.

There are three types of cell death: autophagy, apoptosis, and necrosis. Autophagy is involved in numerous physiological processes in human reproduction, such as normal placental function and homeostasis^11–13^. It is responsible for the degradation of damaged cytoplasmic material in vivo and participates in trophoblast invasion^14^. Nakashima et al*.* showed that impaired autophagy in extravillous trophoblast (EVT) cell lines resulted in the attenuation of invasion capabilities^15^. Suppression of autophagy in trophoblast Rcho-1 cells negatively affected cellular differentiation and invasion activity^16^. Our previous study also found that excessive autophagy induced by glucose oxidase resulted in a significant reduction in trophoblast invasion ability^17^. Mitochondrial autophagy, as a form of intracellular autophagy, is an important regulatory pathway and is closely related to low oxidative stress^18^. Damaged or non-functioning mitochondria are selectively degraded through mitophagy, which is a form of macroautophagy. Both autophagy and mitophagy share similar molecular pathways to form autophagosomes and autolysosomes. In this shared molecular pathway, after LC3-I is transformed into LC3-II, it subsequently relocates and coats on the membrane of autophagosomes. Gen et al. reported that LC3-II coated autophagosomes engulfed degradable substances mediated by p62. Subsequently, the engulfed substances and p62 are degraded by Cathepsin D of the lysosomes in mice^19^. In addition, Beclin-1 was reported to participate in autophagosomes elongation, maturation, and fusion with lysosomes through Beclin-1-containing complex in human lung and kidney cells^20,21^. Furthermore, mitophagy also has its owns specific molecular pathways. The PINK1/Parkin pathway, the mitophagy-specific signaling cascade, can initiate recruitment and activation of the E3 ubiquitin ligase Parkin, and facilitate digestion of damaged mitochondria by autophagosomes^22,23^*. Bc*l-2/adenovirus E1B 19-kDa interacting protein 3 (BNIP3) regulates hypoxia-induced cell death through mitophagy^24^ via direct interaction with the microtubule-associated protein 1A/1B-light chain 3 (LC3) or the recruitment of Parkin^25,26^. Osman et al. reported that BNIP3 could compete for the binding site of Bcl-2 proteins with Beclin-1 to regulate mitophagy in hepatocellular carcinoma^27^. Zhang et.al also reported that BNIP3 could inhibit the degradation of PINK1 to regulate mitophagy in the HEK293 cell line^28^. Interestingly, the expression of BNIP3 in cytotrophoblast cells has been found to be significantly reduced in preeclamptic placentae^29,30^. However, the effect of BNIP3 on PINK1, p62, LC3-II, and Beclin-1 in trophoblast mitophagy and oxidative stress in PE patients remains unclear. We hypothesized that reduced BNIP3 might impair mitophagy through downregulation of LC3-II, Beclin-1, and PINK1 expression, whilst up-regulating p62 expression. In addition, we also evaluated the proliferation, invasion, and migration after BNIP3 knock-down of HTR8/SVneo cells to see if BNIP3 could affect the trophoblast function which is pivotal in the development of PE.

## Results

### Suppressed BNIP3 expression impaired autophagy activities, and led to an accumulation of damaged mitochondria in vivo

**Figure 1:**
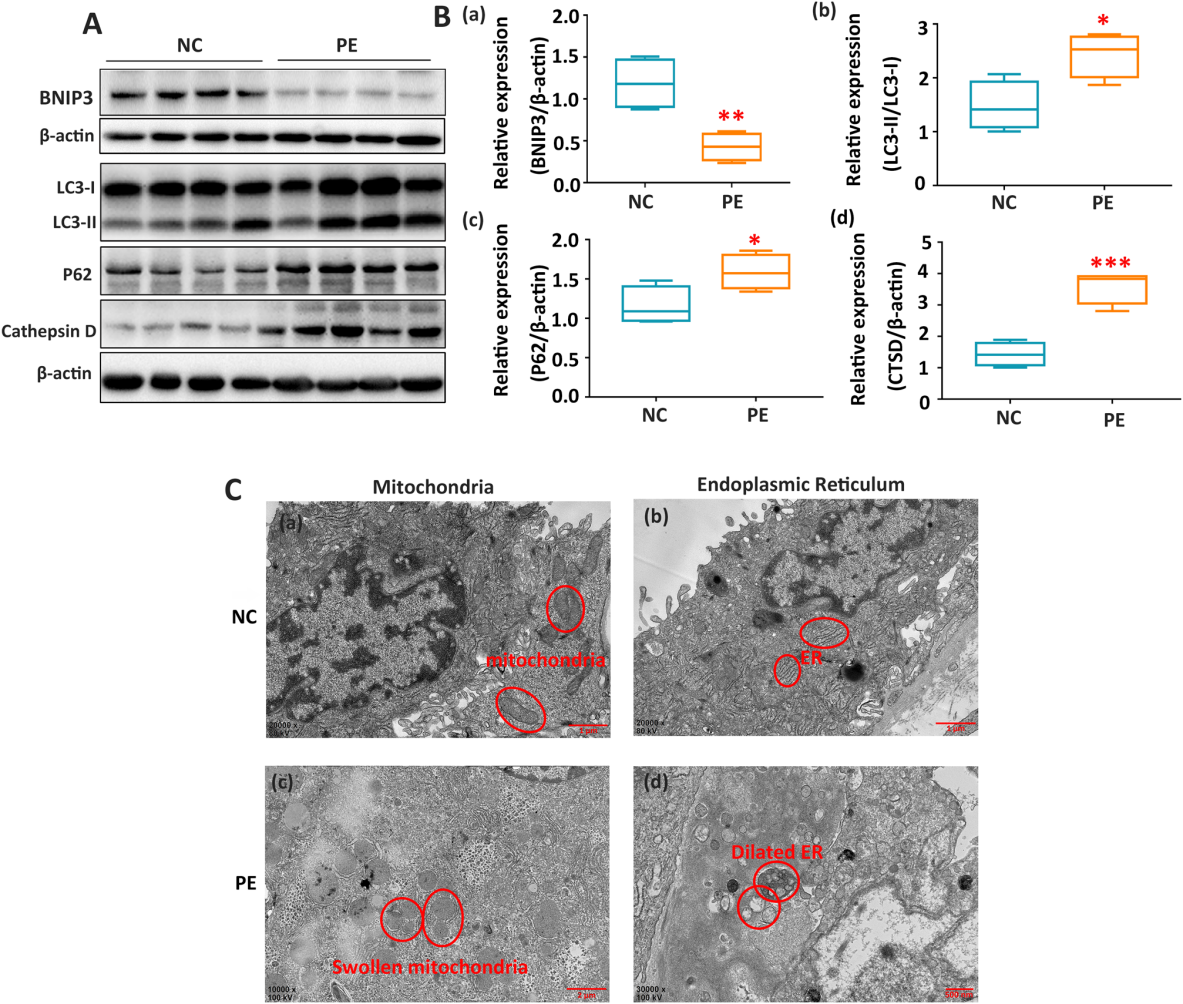
Suppression of BNIP3 with impaired autophagy and increased level of damaged mitochondria in vivo. (**A**,**B**) Western blotting: the proteins in the PE placental tissue that were associated with dysregulation of autophagy were BNIP3, LC3-II, P62, and CTSD for PE (n = 16) and normal pregnancies (n = 18). β-Actin was used as a control *p-value < 0.05, **p-value < 0.01, ***p-value < 0.001. (**C**) Electron micrograph showed the different morphologies of mitochondria and endoplasmic reticulum (indicated by circles) of the placental trophoblast cells from placentae of normal (n = 4) and preeclamptic pregnancies (n = 4). Full-length blots are presented in Supplementary Figure 1.

The expression of BNIP3 was significantly decreased in the PE group when compared with the normal pregnancy group (Fig. 1A,B). Additionally, overexpression of LC3-II, autophagy p62, and Cathepsin D (CTSD) was found in PE placentae when compared with normal placentae (Fig. 1A,B). Results from scanning electron microscopy showed the accumulation of dilated endoplasmic reticulum and swollen mitochondria in PE placentae, which indicated insufficient autophagic activity to recycle damaged mitochondria (Fig. 1C).

### Suppression of BNIP3 expression attenuated autophagosome formation via downregulation of Beclin-1

**Figure 2:**
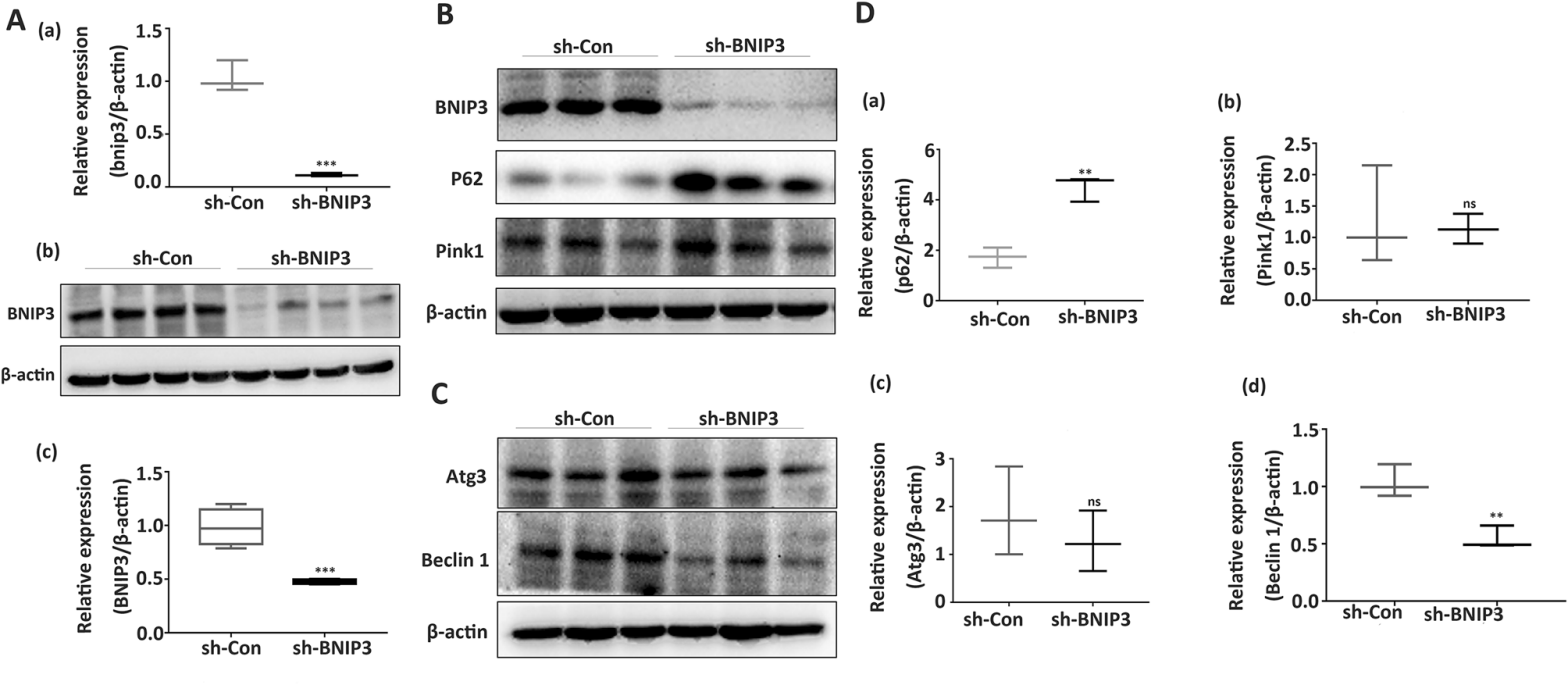
The effects of BNIP3 downregulation on the expression of Beclin-1, P62, Atg3, and Pink1. (**A**) Results from qRT-PCR and Western blot indicated that sh-BNIP3 transfection showed high-efficiency BNIP3 gene silencing (n = 3 per group). (**B**,**D**) Representative Western blot bands of BNIP3, P62, and Pink1 in HTR-8/SVneo cells after sh-RNA transfection. β-Actin was used as a control (n = 3 per group). (**C**,**D**) Representative western blot bands of Beclin-1 and ATG3 in HTR-8/SVneo cells after sh-RNA transfection (n = 3 per group). β-Actin was used as a control. ns: non-significant, *p-value < 0.05, **p-value < 0.01, ***p-value < 0.001. Full-length blots are presented in Supplementary Figure 2.

BNIP3 was successfully silenced in the HTR8/SVneo cell line using lentivirus shRNA against BNIP3, which was supported by the results of qRT-PCR and WB (Fig. 2A(a,b)). The silenced BNIP3 negatively affected autophagy, as p62 was overexpressed in the sh-BNIP3 group (Fig. 2B,D(a)). Downregulation of BNIP3 significantly inhibited the expression of Beclin-1 (Fig. 2B,D(b)) but it had no effect on the expression of Atg3 and Pink1 (Fig. 2C,D(c,d)). These findings indicate that the suppression of BNIP3 and Beclin-1 expression can attenuate mitophagy activities.

### Suppression of BNIP3 expression is associated with reduced oxidative stress-induced mitophagy, ROS accumulation, and apoptosis exacerbation

**Figure 3:**
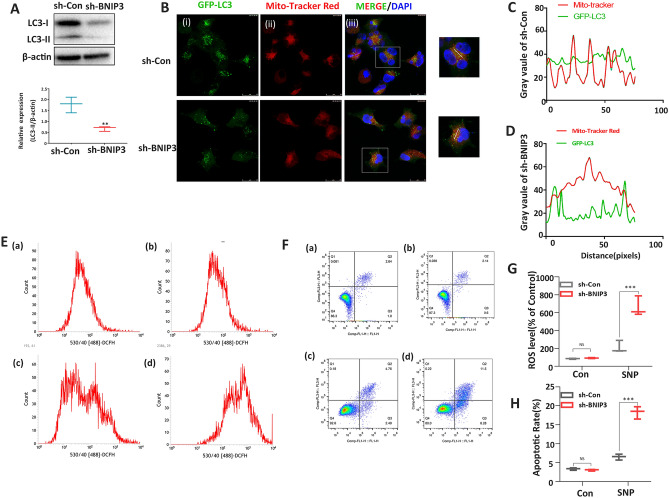
Downregulation of BNIP3 reduced mitophagy, increased the production of reactive oxygen species, and promoted cell death under oxidative stress induced by SNP. (**A**) Relative protein expressions of LC3-I, LC3-II, and β-actin after sh-BNIP3 transfection after SNP treatment (n = 3 per group). (**B**) Immunofluorescent analysis of colocalization of GFP‐LC3 and mitochondria under oxidative stress after sh-Control and sh-BNIP3 transfection (n = 3 per group). (**C**,**D**) Line scans of the co-localizations between GFP-LC3 (Green) and MitoTracker (Red) for sh-Control and sh-BNIP3 under oxidative stress. (**E**) Flow cytometric analysis of intracellular ROS in sh-Control (**a**), sh-BNIP3 (**b**), sh-control + SNP (**c**), and sh-BNIP3 + SNP (**d**) groups (n = 3 per group). (**F**) Flow cytometric analysis of apoptosis in sh-Control (**a**), sh-BNIP3 (**b**), sh-control + SNP (**c**), and sh-BNIP3 + SNP (**d**) groups (n = 3 per group). (**G**) Statistical analysis for flow cytometric analysis of intracellular ROS. (**H**) Statistical analysis for flow cytometric analysis of apoptosis. *NS* non-significant, *p-values < 0 05, **p-value < 0.01, ***p-values < 0.001. Full-length blots are presented in Supplementary Figure 3.

The effect of BNIP3 on cell function under oxidative stress induced by SNP was also investigated in this study. In the WB study, the down-regulation of BNIP3 resulted in underexpression of LC3-II and impaired autophagosome formation (Fig. 3A). In the immunofluorescence line scan analysis, the reduced overlap between LC3 (green) and mitochondria (red) in the sh-BNIP3 group indicate that the colocalization of GFP-LC3 and mitochondria was significantly suppressed in the sh-BNIP3 group (knockdown) under oxidative stress (Fig. 3B–D). Flow cytometry analysis showed an increased level of ROS and a high apoptotic rate in the sh-BNIP3 + SNP group (Fig. 3E–H).

### Suppression of BNIP3 expression inhibited HTR8/SVneo invasion and migration

**Figure 4:**
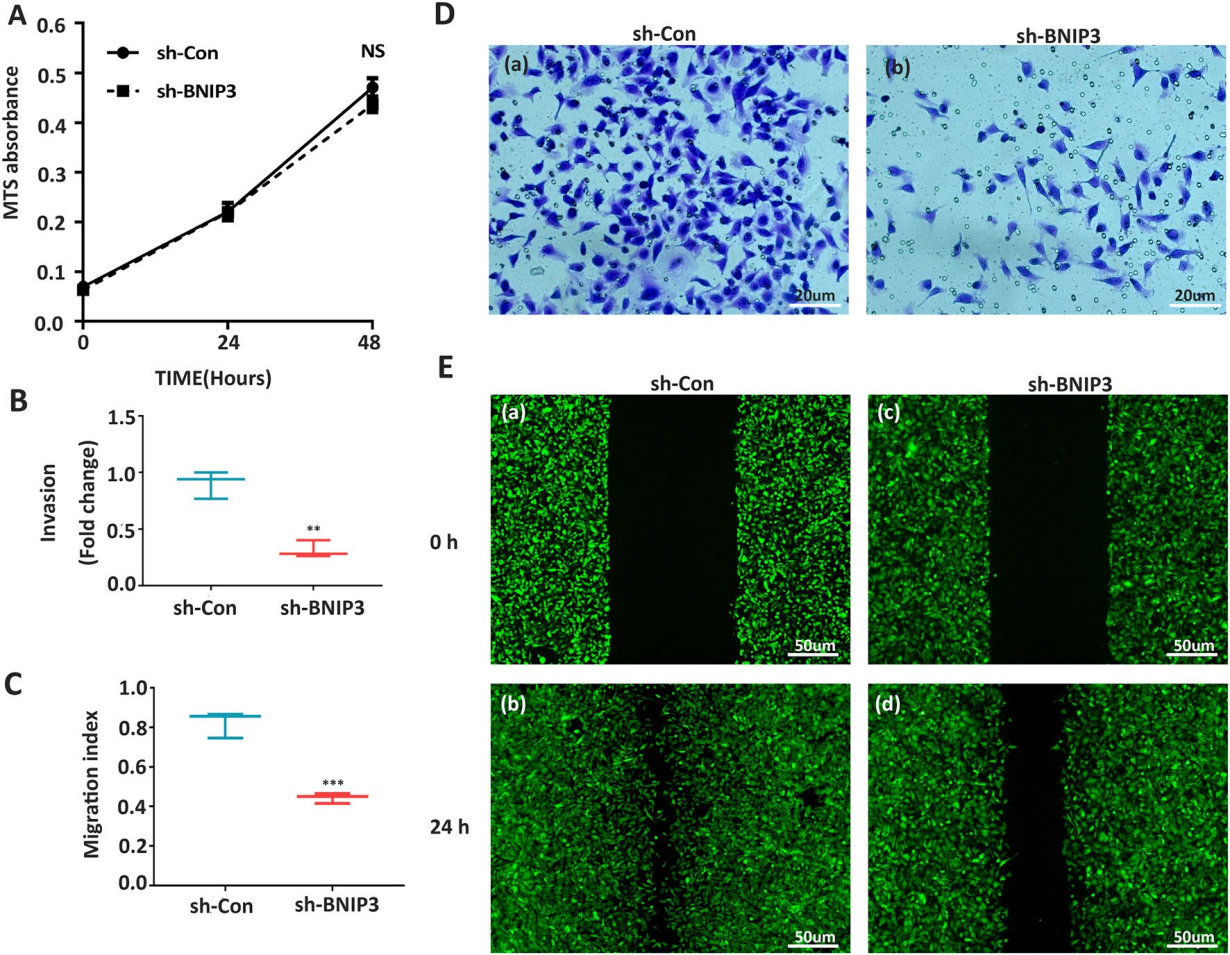
Downregulation of BNIP3 attenuated invasion and migration of trophoblast cells. (**A**) MTS assay on HTR-8/SVneo cells after sh-RNA transfection of 0 h, 24 h, and 48 h (n = 3 per group). (**B**,**D**) Matrigel invasion assays. (**C**,**E**) Wound Healing Assay of HTR-8/SVneo cells after sh-RNA transfection (n = 3 per group). *NS* non-significant, **p-value < 0.01, ***p-value < 0.001).

The MTS cell proliferation assay showed that the downregulation of BNIP3 did not influence cell proliferation (Fig. 4A). However, as compared with the scrambled shRNA group, the trans-well assays showed that suppression of BNIP3 expression attenuated the invasion capability of the HTR8/SVneo cells (Fig. 4B,D). Sh-BNIP3 decreased cell migration significantly when compared with the control group, as assessed using the Wound Healing Assay (Fig. 4C,E).

## Discussion

The present study has demonstrated the effect of knockdown BNIP3 on mitophagy, oxidative stress, apoptosis, and the migration and invasion capabilities of trophoblasts. BNIP3 expression was suppressed along with impaired autophagy and the accumulation of damaged mitochondria in PE placentae. Furthermore, our HTR8/SVneo cell experiments found that the suppression of BNIP3 expression inhibited autophagosome formation and mitophagy, whilst promoting ROS accumulation and apoptosis in an oxidative stress condition. These results suggest that BNIP3 plays multiple roles in the pathophysiology of PE.

**Figure 5:**
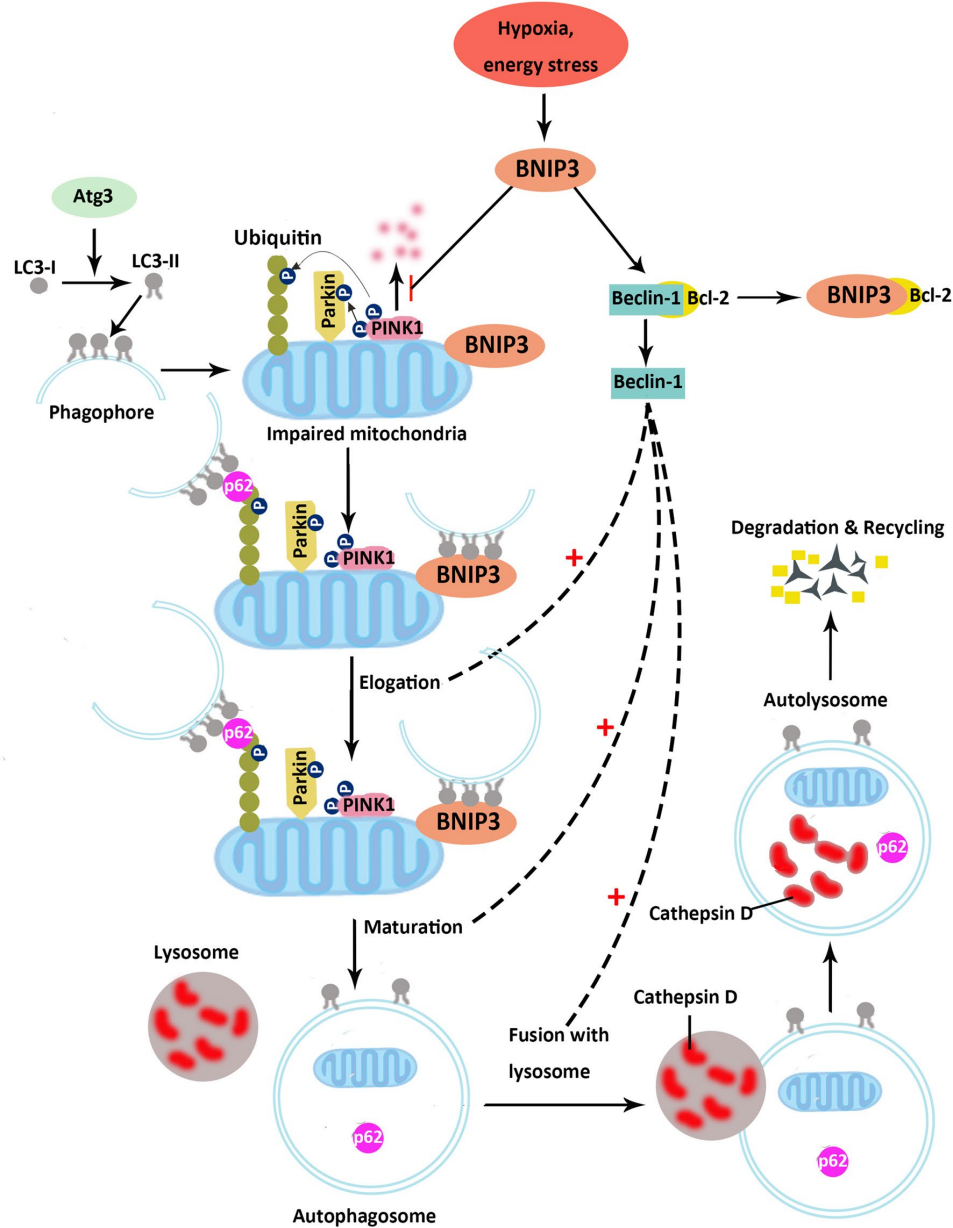
The molecular mechanism of mitophagy. Under hypoxia or energy stress, BNIP3 inhibits the degradation of PINK1 and leads to the accumulation of PINK1 on the outer mitochondrial membrane. PINK1 phosphorylates both Parkin and ubiquitin. Phospho-ubiquitin interacts with LC3-II coated phagophore through p62. BNIP3 can also directly bind with LC3-II and mediate the interaction of phagophores, impairing mitochondrial function. In addition, BNIP3 competes the binding site of Bcl-2 protein with Beclin-1 and releases Beclin-1. The free Beclin-1 subsequently forms the complexes and participates in autophagosome elongation, maturation, and fusion with lysosome. Finally, the impaired mitochondria and p62 are degraded by cathepsin D. (Created by authors using Adobe Illustrator CS6).

Autophagy is an essential adaptive response to exogenous stimuli such as hypoxia, nutritional deficiency, and pathogenic infection^31,32^. We found that dysregulation of BNIP3 and impairment of autophagy coexisted in PE placentae in the present study. In agreement with other studies, the expression of BNIP3 was reduced in PE patients^33,34^. However, a study from Xu et al. found upregulation of the BNIP3 protein with mitochondrion disassembly in the placentae of patients with early-onset PE^35^. These inconsistent results might be explained by the use of placentae of different gestational ages. There was a four week difference in gestational age between the healthy pregnancy group and PE group in Xu et al.’s study. However, there was little difference in gestational age between the healthy pregnancy group and PE group in the present study. The expression of p62 has also previously been reported to be enhanced in human preeclamptic placental EVT cells (cytokeratin 7-positive) when compared with normotensive females^15^. The same finding was observed in the present study; p62 was overexpressed when BNIP3 was suppressed in PE patients and as a result, autophagy was inhibited. In agreement with a study of the human umbilical vein endothelial cell line, the knockdown of BNIP3 significantly reduced the expression of LC3-I and LC3-II, which are protein markers for autophagy^36^. However, we found the expression of LC3-II to be increased in the placenta of PE patients. The reason for the discrepancy might be that the HTR8/SVneo cell line is mainly used for investigating the effect of suppressed BNIP3 in early pregnancy, while the placentas we collected were at the terminal stage of PE. LC3 was also reported as a regulator of apoptosis-extrinsic pathway and accumulation of LC3 could activate apoptosis^37,38^. Thus, we speculated that suppressed BNIP3 impairs mitophagy in early pregnancy via downregulation of LC3-II and subsequently lead to the accumulation of impaired mitochondria and increased apoptosis (which may be reflected by the LC3-II overexpression) in late PE pregnancy. The proposed mechanism to explain the interaction between LC3-II, p62, and cathepsin D and their involvement in macrophage impairment is illustrated in Fig. 5. To summarize, these findings support the notion that the reduction of BNIP3 leads to impaired autophagosome formation because p62 could be transported into the phagophore membrane to form autophagosomes^39^ and LC3-II locates at autophagosomes to regulate autophagy^25^.

To further investigate the influence of BNIP3 on autophagosome formation, the three associated proteins, i.e., Beclin-1, Pink1, and Atg3, were measured in BNIP3-knockdown trophoblast cells in this study. We found that the downregulation of BNIP3 resulted in a substantial reduction in Beclin-1 (which has also been found previously^40^), while it had no influence on the expression of Pink1 and Atg3. The Beclin 1-Vps34 protein–protein interaction network is critical for autophagy regulation. This complex ensures the elongation of phagophores and therefore the ability to seal off to form mature autophagosomes^41^. In hypoxia, high levels of BNIP3 displace Beclin1 from Bcl-xL and Bcl-2, leading to autophagy^42,43^. Knockdown BNIP3 directly reduced the free Beclin-1 and subsequently impaired autophagosome formation. However, PINK1 and Atg3 were not affected by the level of BNIP3. The reasons for this observation are unknown.

It has been found that mitophagy can remove impaired mitochondria to reduce excessive ROS and ensure an optimal balance between mitochondrial biogenesis and turnover^44^. Mitophagy appeared to be impaired in PE placentae in our study, and varying degrees of mitochondrial damage was observed in the trophoblasts of PE patients. The results of the colocalization of mitochondria and GFP-LC3 showed a significant reduction after BNIP3 was knocked down, which was an indicator of mitophagy impairment. In mammals with BNIP3 knockdown, hypoxic conditions could reduce mitochondrial function dramatically^45^.

Oxidative stress is one of the major factors known to be associated with PE, and mitochondrial ROS generation has been found to be an important source of oxidative stress in PE^46^. BNIP3 is essential to maintain ROS homeostasis^43^ and the flow cytometry analysis in our study showed that when BNIP3 was suppressed, ROS was increased and accumulated in cells. In agreement with previous studies, we observed deficiencies in the antioxidant defenses and an abnormally high generation of ROS in the placental mitochondria from PE patients^47,48^.

Of the PE therapies under investigation, mitochondrial antioxidant targeting has been identified as one of the most promising strategies. An in vitro study found that mitochondrial-targeted antioxidants significantly reduced mitochondrial ROS production and inflammatory profiles in a PE cell model^49^. Mitochondrial antioxidants (e.g. MitoQ/MitoTEMPO) significantly improved blood pressure and fetal weights in a rat model with reduced uterine perfusion pressure, which suggested that targeting mitochondrial ROS could be a therapeutic success^48^.

It is apparent that overwhelming oxidative stress causes widespread destruction of cellular macromolecules (lipids, proteins, DNA, etc.) and organelles, subsequently leading to autophagy. In the early stage of pregnancy, before vascular remodeling, the placenta grows in a low-oxygen environment, which is susceptible to oxidative damage^50,51^. Oxidative stress and the systemic inflammatory response were observed at a much greater degree in the knockdown BNIP3 group, as the synthesis of autophagosomes was impaired. The positive amplification of oxidative damage ultimately leads to cell death. In addition, down-regulated BNIP3 might influence the invasion and migration of trophoblasts. Since trophoblasts invade the endometrium to ensure successful vascular remodeling in early pregnancy, our results showed that suppressed BNIP3 might lead to PE through the decreased invasion ability of trophoblasts. There is a degree of similarity between the metastatic process and the invasion of endometrium by trophoblasts^52^. A study has shown that BNIP3-deficient cells by methylation remarkably upregulated cancer cell migration and invasion in hypoxia^53^. Future work could focus on the epigenetic regulation of BNIP3 expression, such as methylation, deacetylation, and ubiquitination^54^.

In summary, the dysregulated mitophagy observed in PE patients could be due to the suppression of BNIP3 and deficiencies in the antioxidant defenses; the association between BNIP3 and mitophagy in the development of PE is of clinical importance.

## Materials and methods

### Study participants and selection criteria

This study was approved by the ethics committee of Chongqing Medical University, Chongqing, China (Ethic No. 2014034) and each participant provided written informed consent. The study was performed in accordance with the Declaration of Helsinki. A total of 34 participants, including 16 participants with a diagnosis of early-onset PE and 18 normal participants, were recruited from the Department of Gynecology and Obstetrics, The First Affiliated Hospital of Chongqing Medical University, Chongqing, China. All participants underwent cesarean delivery.

The diagnosis of PE was based on the American Congress of Obstetricians and Gynecologists guidelines^55^. PE is characterized by systolic blood pressure exceeding 160 mmHg and/or diastolic blood pressure exceeding 110 mmHg in more than one reading separated by at least 6 h, in conjunction with proteinuria. It is important to note that all our PE participants were diagnosed before 34 weeks of gestation, which is the diagnosis criteria for early-onset PE^56^. Patients with other pregnancy complications or chronic medical disorders, such as gestational diabetes mellitus, preterm birth, fetal growth restriction, multiple pregnancies, cardiovascular disease, collagen disorder, chronic renal disease, chronic hypertension, and other metabolic diseases were excluded from the study. The detailed clinical data of participants is displayed in Table 1.

**Table 1:** Clinical characteristics of the participants.

	PE (n = 16)	Normal (n = 18)	p value
Age (years)	29 (27.3, 32.0)	30 (27.8, 33.3)	0.574^b^
BMI at delivery (kg/m^2^)	29.9 (28.7, 32.2)	29.6 (28.4, 30.4)	0.224^b^
Gestational age (weeks)	36.6 (35.2, 38.3)	37.6 (37.5, 38.2)	0.046^b^
Parity	0 (0, 1)	0 (0, 1)	0.878^b^
Systolic blood pressure (mmHg)	169.5 (163.3, 180.0)	115 (110.8, 119.5)	< 0.0001^b^
Diastolic blood pressure (mmHg)	107.5 (100.5, 113.8)	68 (66.8, 69.3)	< 0.0001^b^
Proteinuria	3 (3, 1)	0 (0, 1)	< 0.0001^b^
Placental weight (g)	480 (430, 500)	545 (517, 600)	< 0.0001^b^
Neonatal birth weight (g)	2383.1 ± 320.3	3456.1 ± 224.4	< 0.001^a^
Ratio of the newborns (male/female)	8/8	8/10	0.746^c^
Smoking history	None	None	

*PE* pre-eclampsia, *BMI* body mass index; ^a^Student *t* test; ^b^Mann–Whitney test; ^c^Chi-squared test.

### Human placental tissue collection

The placental tissues were collected after delivery and transferred immediately into cold phosphate buffer saline (PBS) on ice, as described previously^57^. Briefly, after removal of the basal plate, placental tissue samples were obtained from 10 placental cotyledons without visible calcification, infarction, hematoma, or tears to ensure a representative sample was obtained. The tissue samples were then washed with ice-cold PBS, frozen by liquid nitrogen, and stored at − 80 °C.

### Transmission electron microscopy

Placental tissues (1 mm^3^/piece, five pieces from each placenta) were fixed in 4% glutaraldehyde at 4 °C overnight then underwent ultrathin sectioning. Images were captured and processed using a Hitachi-7500 transmission electron microscope (Hitachi Limited, Japan).

### Cell culture

The human transformed EVT cell line, HTR8/SVneo, was provided by Dr. C. H. Graham (Queen’s University, Kingston, ON, Canada). The cells were incubated in a medium with 89% RPMI 1640 medium (Gibco, USA), 10% fetal bovine serum (Gibco, USA), and 1% penicillin + streptomycin mixture at 37 °C in a humidified atmosphere with 5% CO_2_.

### Cell treatments

Cells were treated with short hairpin (sh) RNA via sh-BNIP3 (sh-BNIP3: 5′-GAACUGCACUUCAGCAAUATT-3′) or sh-Control lentivirus (GenePharma, Shanghai China) for 48 h. The uninfected cells were eliminated by hygromycin at a concentration of 200 μg/ml. All the selected cells were then transfected by lentivirus short hairpin RNA (shRNA) against GFP-tagged LC3 (GenePharma, Shanghai, China) to link the green fluorescent proteins with the LC3 protein, then to mark LC3 and colocalize the mitochondria. The uninfected cells were eliminated by puromycin at a concentration of 2 μg/ml. To establish the oxidative stress cell model, the cells were treated with 5 μm sodium nitroprusside (SNP) for 8 h, as previously described^9,58^. There were four groups in this study: (1) sh-BNIP3; (2) sh-Control; (3) sh-BNIP3 + SNP; and (4) sh-Control + SNP.

### Immunofluorescence

Mitochondria were labeled using MitoTracker™ Deep Red FM (Thermo-fisher, USA) and LC3 were labeled using green fluorescent proteins, before being fixed with paraformaldehyde at room temperature. After fixation, they were mounted by antifade mounting medium with DAPI (H-1200, Vector Laboratories, Burlingame, CA, USA). The slides were imaged using a laser confocal microscope (Leica, Wetzlar, Germany). The colocalization of GFP-LC3 and mitochondria was analyzed using Image J and the results were drawn as line graphs using Graph-Pad Prism software (GraphPad Software, California, USA).

### Matrigel cell invasion assay

The matrigel invasion chamber was used to evaluate the impact of BNIP3 knockdown on cell invasion. The cell inserts (Costar, USA) were coated with Matrigel (BD BioScience, USA) and placed within a 24-well plate. In total, 3 × 10^5^ cells per group were resuspended into 200 μl of RPMI-1640 medium containing 1% Fetal Bovine Serum (FBS). They were added into a transwell chamber. The chamber was placed into a 24-well plate filled with 600 μl RPMI-1640 medium that contained 10% FBS. The cells were incubated at 37 °C with 5% CO_2_. After 24 h, the transwell chambers were removed and washed with PBS then fixed with methanol and stained with 0.1% crystal violet (Beyotime Biotechnology, China). The number of migratory cells was counted using Image J.

### Wound healing assay

In total, 1 × 10^5^ cells of sh-BNIP3 and sh-Control were inoculated into six-well plates. When the cell density reached 80%, the cellular monolayer was scratched using a 1 ml clean pipette tip. The cells were then gently washed with PBS three times. This time point was defined as time 0 h. The migration was imaged at 0 h and 24 h, and analyzed using Image J.

### Quantitative real-time PCR (qRT-PCR)

The RNAiso Plus (Takara Bio, 9108) was used to extract total RNA and the subsequential reverse transcription was conducted with a PrimeScript™ RT reagent Kit (Takara Bio, RR047A). The resulting cDNAs or DNAs were quantified using real-time PCR with a SYBR PrimeScript PLUS RT-PCR Kit (Takara Bio, RR096A) on the Agilent Mx3000P Real-Time PCR System (Agilent Technologies, Santa Clara, CA, USA), using the following primers: BNIP3 forward primer, 5′-ACCACAAGATACCAACAGAG-3′; BNIP3 reverse primer, 5′-AGAGCAGCAGAGATGGAA-3′; β-actin forward primer, 5′-CTGTGCTATGTTGCCCTGGACTTC-3′; β-actin reverse primer, 5′-GCTCGTTGCCGATGGTGATGAC-3′. The relative expression was calculated using the 2-ΔΔCT method.

### Western blot (WB)

Frozen placental tissues and cultured cells were suspended with RIPA lysis buffer for protein extraction. A BCA Protein Assay Kit (Beyotime Biotechnology, China) was used to quantify protein concentrations. Protein samples were separated by SDS polyacrylamide gels and transferred to polyvinylidene difluoride membranes (Millipore, USA). 5% nonfat dry milk (Bio-Rad, California, USA) was used for nonspecific protein band blocking, and the results were quantified using a ChemiDoc™ XRS + (Bio-Rad, USA). The antibodies used in WB were anti-BNIP3 (1:1000, Abcam, Catalog#: ab109362), ATG3 (1:500, Santa Cruz, Catalog#: sc-393660), Beclin-1 (1:500, Santa Cruz, Catalog#: sc-48341), P62 (1:500, Santa Cruz, Catalog#: sc-25329), β-actin (1:2000, ZSGB-BIO, Catalog#: TA-09), LC3 (1:500; NOVUS, Catalog#: NB100-2220), PINK 1 (1:500, Santa Cruz, Catalog#: sc-517353) and Cathepsin D (1:500, Wanleibio, Catalog#: WL01234).

### Flow cytometry analysis

Flow cytometry (BD Biosciences, USA) was used to quantify the reactive oxygen species (ROS) production and apoptotic rate of the cells. The production of ROS was measured using a ROS Assay Kit (Beyotime Biotechnology, China) according to the manufacturer’s instructions. After cells were treated with SNP for 8 h and washed with PBS, DCFH-DA was added, and the cells were incubated for 20 min at 37 °C. The cells were then isolated by centrifugation (800 rpm for 3 min) and resuspended with cold PBS before the ROS were measured. At the same time, the apoptotic rate was measured using an Annexin V-FITC/PI Apoptosis Detection Kit (Vazyme, China) according to the manufacturer’s instructions. After the treatment of SNP and centrifugation, cells were incubated with Annexin V-FITC/PI for 20 min in a dark room at room temperature. Subsequently, the apoptotic rate of the treated cells was measured by flow cytometry (BD Biosciences, USA).

### Cell viability assay

The viability of the cells was measured using the MTS Cell Proliferation Colorimetric Assay Kit. 5 × 10^5^ cells from the sh-Control and sh-BNIP3 groups were seeded into a 96-cell plate and cultured for 24 h and 48 h respectively. After incubation, the MTS reagent was added and the cells were incubated at 37 °C for 1 h. The supernatant was collected after centrifugation and the cell viability was determined using a spectrophotometer (brand) at 495 nm.

### Statistical analysis

Data were coded then analyzed by the Graph-Pad Prism software (GraphPad Software, California, USA). Continuous variables were expressed as the mean ± standard deviation (SD) if normally distributed, or as the median and interquartile range (IQR) if they had a skewed distribution. Categorical variables were described as counts and percentages. The Student t-test, Mann–Whitney test, and Chi-squared test were used to test for significant differences between groups. A *p*-value of < 0.05 was considered to be statistically significant.

## Supplementary Information

Supplementary Figure 1.

Supplementary Figure 2.

Supplementary Figure 3.

## Data Availability

All data generated or analysed during this study are included in this published article and its Supplementary Information files.

## References

[1] Steegers EA, von Dadelszen P, Duvekot JJ, Pijnenborg R (2010). Pre-eclampsia. Lancet.

[2] Abalos E, Cuesta C, Grosso AL, Chou D, Say L (2013). Global and regional estimates of preeclampsia and eclampsia: A systematic review. Eur. J. Obstet. Gynecol. Reprod. Biol..

[3] Mol BWJ (2016). Pre-eclampsia. Lancet.

[4] Smith GC, Pell JP, Walsh D (2001). Pregnancy complications and maternal risk of ischaemic heart disease: A retrospective cohort study of 129,290 births. Lancet.

[5] Bell MJ (2010). A historical overview of preeclampsia-eclampsia. J. Obstet. Gynecol. Neonatal Nurs..

[6] Saito S, Nakashima A (2014). A review of the mechanism for poor placentation in early-onset preeclampsia: The role of autophagy in trophoblast invasion and vascular remodeling. J. Reprod. Immunol..

[7] Garrido-Gomez T (2017). Defective decidualization during and after severe preeclampsia reveals a possible maternal contribution to the etiology. Proc. Natl. Acad. Sci. U.S.A..

[8] Kalkat M (2013). Placental autophagy regulation by the BOK-MCL1 rheostat. Autophagy.

[9] Melland-Smith M (2015). Disruption of sphingolipid metabolism augments ceramide-induced autophagy in preeclampsia. Autophagy.

[10] Longtine MS, Chen B, Odibo AO, Zhong Y, Nelson DM (2012). Villous trophoblast apoptosis is elevated and restricted to cytotrophoblasts in pregnancies complicated by preeclampsia, IUGR, or preeclampsia with IUGR. Placenta.

[11] Mizushima N, Komatsu M (2011). Autophagy: Renovation of cells and tissues. Cell.

[12] Zhao X (2020). Physiological and pathological regulation of autophagy in pregnancy. Arch. Gynecol. Obstet..

[13] Sato M, Sato K (2013). Dynamic regulation of autophagy and endocytosis for cell remodeling during early development. Traffic.

[14] Oh SY (2020). Autophagy regulates trophoblast invasion by targeting NF-kappaB activity. Sci. Rep..

[15] Nakashima A (2013). Impaired autophagy by soluble endoglin, under physiological hypoxia in early pregnant period, is involved in poor placentation in preeclampsia. Autophagy.

[16] Arikawa T (2016). Galectin-4 expression is down-regulated in response to autophagy during differentiation of rat trophoblast cells. Sci. Rep..

[17] Gao L (2015). Excessive autophagy induces the failure of trophoblast invasion and vasculature: Possible relevance to the pathogenesis of preeclampsia. J. Hypertens..

[18] Katayama H (2020). Visualizing and modulating mitophagy for therapeutic studies of neurodegeneration. Cell.

[19] Matsumoto G, Wada K, Okuno M, Kurosawa M, Nukina N (2011). Serine 403 phosphorylation of p62/SQSTM1 regulates selective autophagic clearance of ubiquitinated proteins. Mol. Cell.

[20] Matsunaga K (2009). Two Beclin 1-binding proteins, Atg14L and Rubicon, reciprocally regulate autophagy at different stages. Nat. Cell Biol..

[21] Liang C (2006). Autophagic and tumour suppressor activity of a novel Beclin1-binding protein UVRAG. Nat. Cell Biol..

[22] Panigrahi DP (2019). The emerging, multifaceted role of mitophagy in cancer and cancer therapeutics. Semin. Cancer Biol..

[23] Lin Q (2019). PINK1-parkin pathway of mitophagy protects against contrast-induced acute kidney injury via decreasing mitochondrial ROS and NLRP3 inflammasome activation. Redox Biol..

[24] Pickles S, Vigie P, Youle RJ (2018). Mitophagy and quality control mechanisms in mitochondrial maintenance. Curr. Biol..

[25] Yu D (2018). Bcl-2/E1B-19KD-interacting protein 3/light chain 3 interaction induces mitophagy in spinal cord injury in rats both in vivo and in vitro. J. Neurotrauma.

[26] Lee Y, Lee HY, Hanna RA, Gustafsson AB (2011). Mitochondrial autophagy by Bnip3 involves Drp1-mediated mitochondrial fission and recruitment of Parkin in cardiac myocytes. Am. J. Physiol. Heart Circ. Physiol..

[27] Osman NA, Abd El-Rehim DM, Kamal IM (2015). Defective Beclin-1 and elevated hypoxia-inducible factor (HIF)-1alpha expression are closely linked to tumorigenesis, differentiation, and progression of hepatocellular carcinoma. Tumour Biol..

[28] Zhang T (2016). BNIP3 protein suppresses PINK1 kinase proteolytic cleavage to promote mitophagy. J. Biol. Chem..

[29] Stepan H, Leo C, Purz S, Hockel M, Horn LC (2005). Placental localization and expression of the cell death factors BNip3 and Nix in preeclampsia, intrauterine growth retardation and HELLP syndrome. Eur. J. Obstet. Gynecol. Reprod. Biol..

[30] Zhou X (2017). Impaired mitochondrial fusion, autophagy, biogenesis and dysregulated lipid metabolism is associated with preeclampsia. Exp. Cell Res..

[31] Nowosad A (2020). p27 controls Ragulator and mTOR activity in amino acid-deprived cells to regulate the autophagy-lysosomal pathway and coordinate cell cycle and cell growth. Nat. Cell Biol..

[32] Kroemer G, Marino G, Levine B (2010). Autophagy and the integrated stress response. Mol. Cell.

[33] Tong J (2018). Transcriptomic profiling in human decidua of severe preeclampsia detected by RNA sequencing. J. Cell Biochem..

[34] Ma J (2019). Dysfunction of B-cell lymphoma 2/adenovirus E1B 19KD interacting protein 3 in decidua is involved in the pathogenesis of preeclampsia. J. Hypertens..

[35] Xu Z (2018). Proteomics analysis reveals abnormal electron transport and excessive oxidative stress cause mitochondrial dysfunction in placental tissues of early-onset preeclampsia. Proteomics Clin. Appl..

[36] Filippi I (2018). Different adaptive responses to hypoxia in normal and multiple myeloma endothelial cells. Cell Physiol. Biochem..

[37] Wang K (2015). Autophagy and apoptosis in liver injury. Cell Cycle.

[38] Xu D (2019). SIRT2 functions in aging, autophagy, and apoptosis in post-maturation bovine oocytes. Life Sci..

[39] Lim J (2015). Proteotoxic stress induces phosphorylation of p62/SQSTM1 by ULK1 to regulate selective autophagic clearance of protein aggregates. PLoS Genet..

[40] Bellot G (2009). Hypoxia-induced autophagy is mediated through hypoxia-inducible factor induction of BNIP3 and BNIP3L via their BH3 domains. Mol. Cell Biol..

[41] Morris DH, Yip CK, Shi Y, Chait BT, Wang QJ (2015). Beclin 1-Vps34 complex architecture: Understanding the nuts and bolts of therapeutic targets. Front. Biol. (Beijing).

[42] Liu XW, Lu MK, Zhong HT, Wang LH, Fu YP (2019). *Panax notoginseng* saponins attenuate myocardial ischemia-reperfusion injury through the HIF-1alpha/BNIP3 pathway of autophagy. J. Cardiovasc. Pharmacol..

[43] Burton TR, Gibson SB (2009). The role of Bcl-2 family member BNIP3 in cell death and disease: NIPping at the heels of cell death. Cell Death Differ..

[44] Larson-Casey JL, Deshane JS, Ryan AJ, Thannickal VJ, Carter AB (2016). Macrophage Akt1 kinase-mediated mitophagy modulates apoptosis resistance and pulmonary fibrosis. Immunity.

[45] Ney PA (1853). Mitochondrial autophagy: Origins, significance, and role of BNIP3 and NIX. Biochim. Biophys. Acta.

[46] Wang Y, Walsh SW (1998). Placental mitochondria as a source of oxidative stress in pre-eclampsia. Placenta.

[47] D'Souza V (2016). Increased oxidative stress from early pregnancy in women who develop preeclampsia. Clin. Exp. Hypertens..

[48] Vaka VR (2018). Role of mitochondrial dysfunction and reactive oxygen species in mediating hypertension in the reduced uterine perfusion pressure rat model of preeclampsia. Hypertension.

[49] McCarthy C, Kenny LC (2016). Therapeutically targeting mitochondrial redox signalling alleviates endothelial dysfunction in preeclampsia. Sci. Rep..

[50] Genbacev O, Zhou Y, Ludlow JW, Fisher SJ (1997). Regulation of human placental development by oxygen tension. Science.

[51] Jauniaux E, Watson A, Burton G (2001). Evaluation of respiratory gases and acid-base gradients in human fetal fluids and uteroplacental tissue between 7 and 16 weeks' gestation. Am. J. Obstet. Gynecol..

[52] Knofler M, Pollheimer J (2012). IFPA Award in Placentology lecture: Molecular regulation of human trophoblast invasion. Placenta.

[53] Kamino H (2016). Mieap-regulated mitochondrial quality control is frequently inactivated in human colorectal cancer. Oncogenesis.

[54] Baek SH, Kim KI (2017). Epigenetic control of autophagy: Nuclear events gain more attention. Mol. Cell.

[55] ACOG Committee on Practice Bulletins-Obstetrics (2002). Diagnosis and management of preeclampsia and eclampsia. Number 33, January 2002. Obstet. Gynecol..

[56] von Dadelszen P, Magee LA, Roberts JM (2003). Subclassification of preeclampsia. Hypertens. Pregnancy.

[57] Pasupathy D (2008). Study protocol. A prospective cohort study of unselected primiparous women: The pregnancy outcome prediction study. BMC Pregnancy Childbirth.

[58] Chen H (2018). Decreased IL-33 production contributes to trophoblast cell dysfunction in pregnancies with preeclampsia. Mediat. Inflamm..

