# Supplementary figures and images for: Impaired placental mitophagy and oxidative stress are associated with dysregulated BNIP3 in preeclampsia
Source: Sci Rep. 2021 Oct 14;11:20469. doi: 10.1038/s41598-021-99837-1 (PMC8516954; doi:10.1038/s41598-021-99837-1)

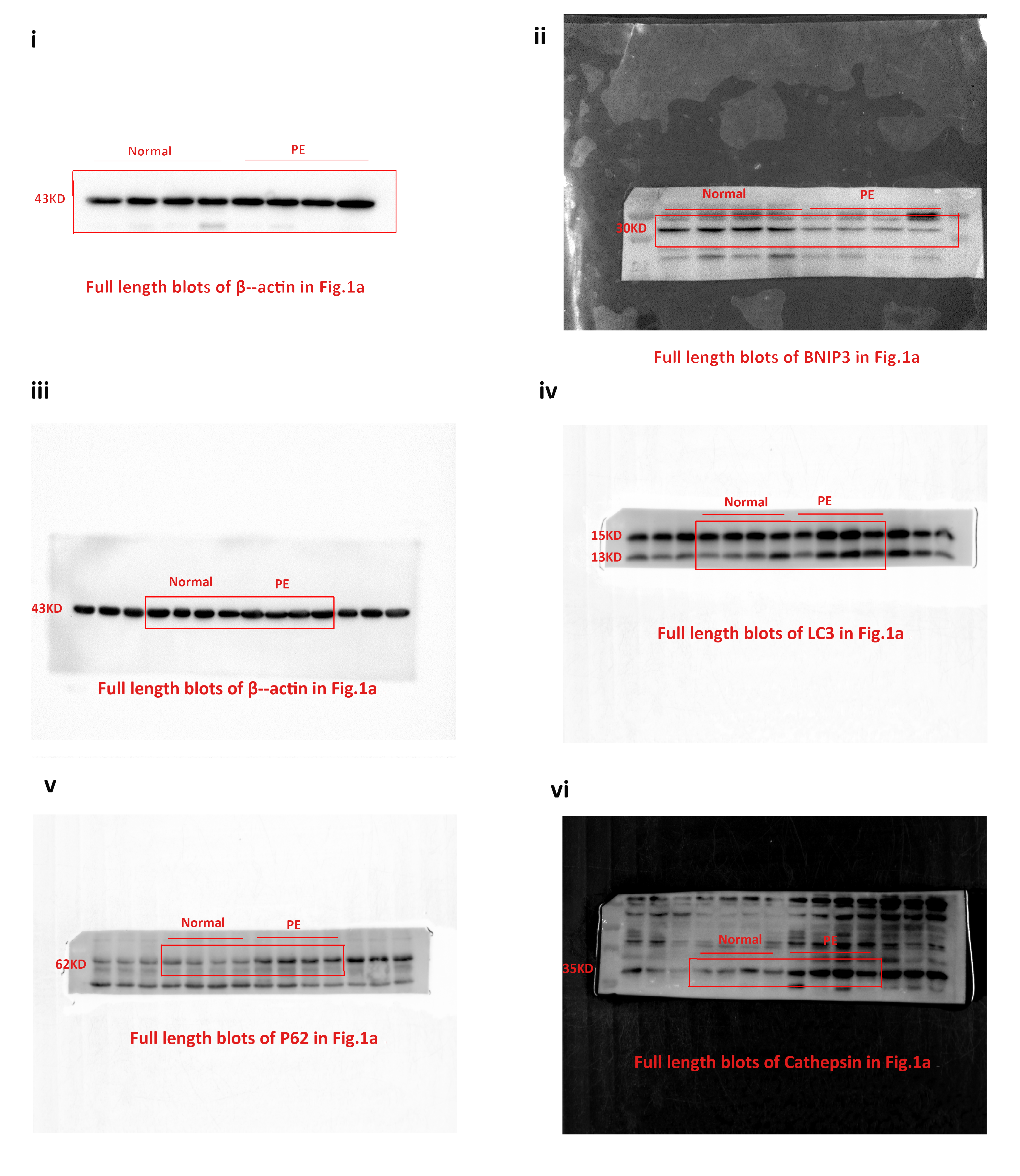

Supplement: Supplementary file 1 — Supplementary Figure 1. [file 41598_2021_99837_MOESM1_ESM.tif]

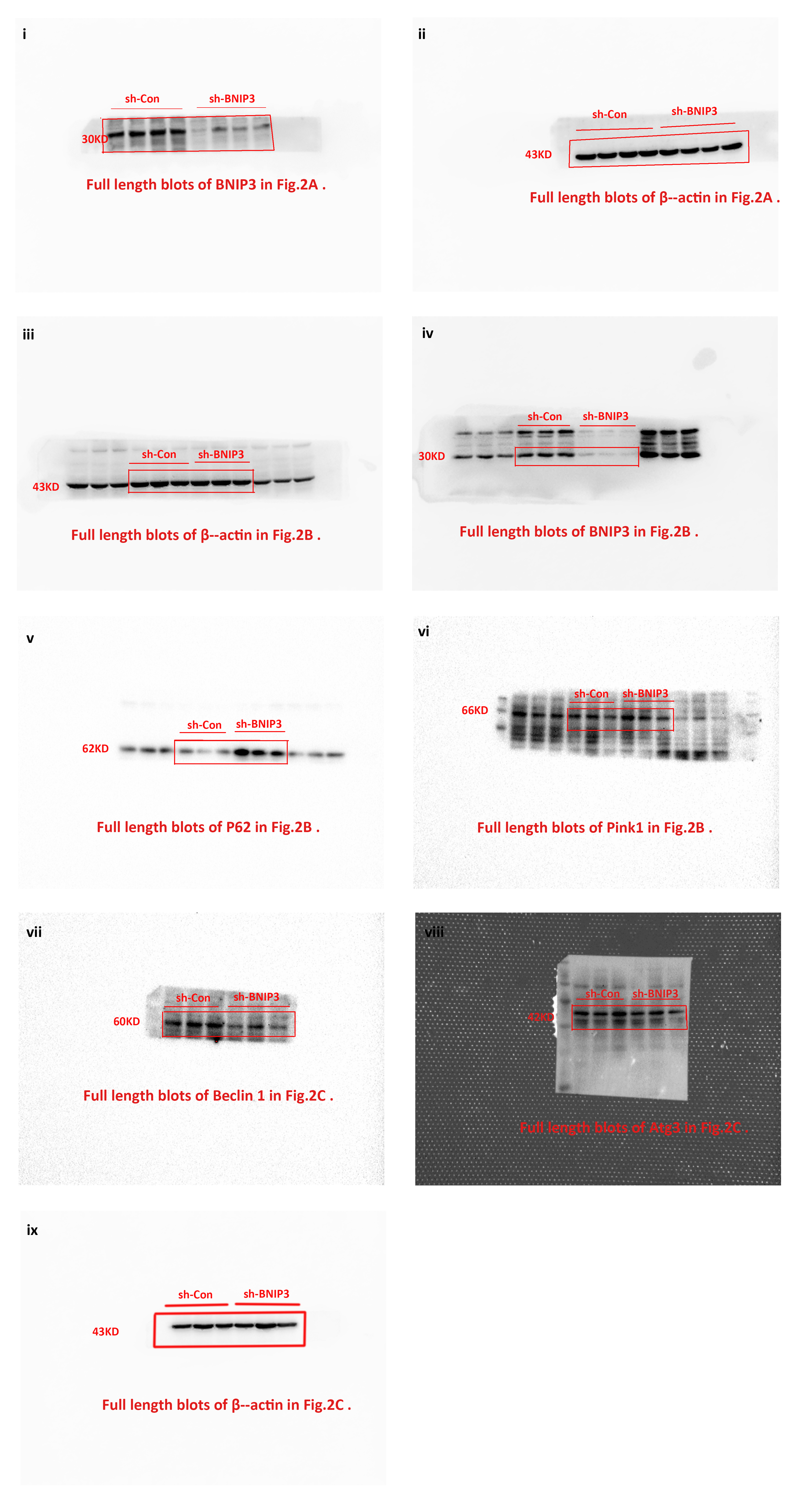

Supplement: Supplementary file 2 — Supplementary Figure 2. [file 41598_2021_99837_MOESM2_ESM.tif]

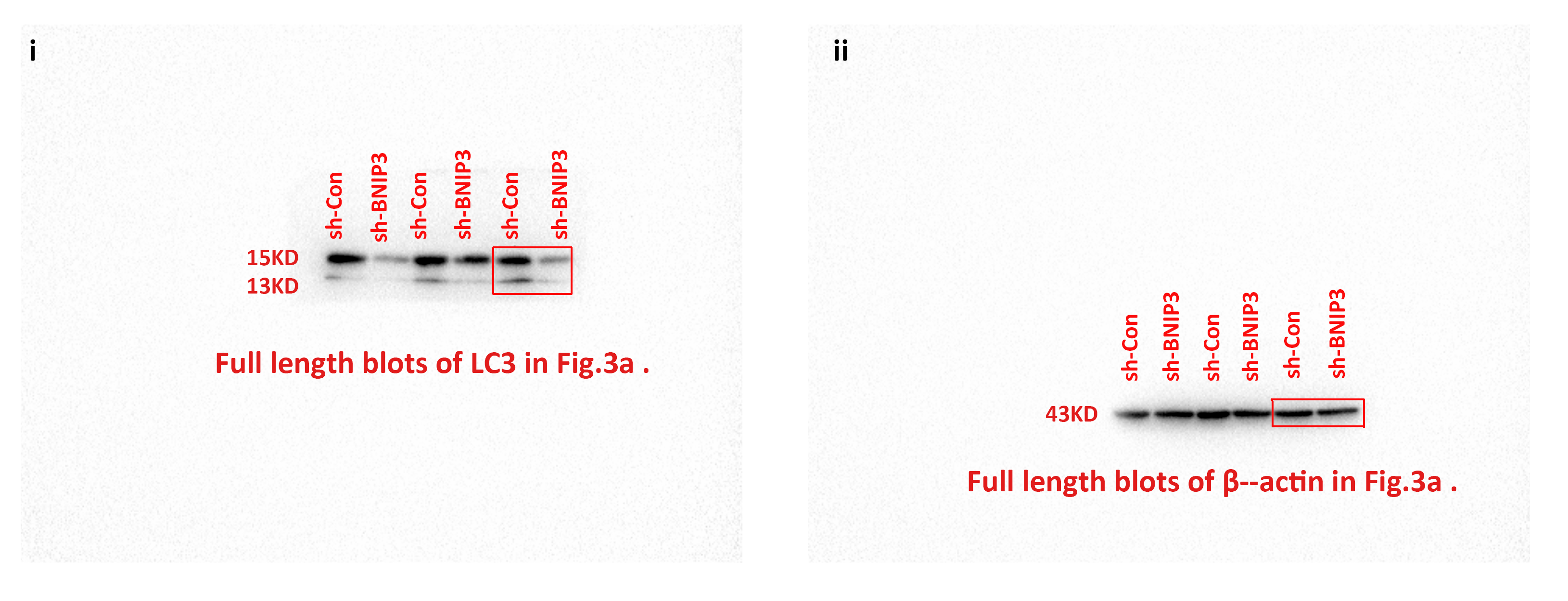

Supplement: Supplementary file 3 — Supplementary Figure 3. [file 41598_2021_99837_MOESM3_ESM.tif]
